# Solvent-Free Microwave-Assisted Extraction of Polyphenols from Olive Tree Leaves: Antioxidant and Antimicrobial Properties

**DOI:** 10.3390/molecules22071056

**Published:** 2017-06-24

**Authors:** Selin Şahin, Ruya Samli, Ayşe Seher Birteksöz Tan, Francisco J. Barba, Farid Chemat, Giancarlo Cravotto, José M. Lorenzo

**Affiliations:** 1 Department of Chemical Engineering, Engineering Faculty, Istanbul University, 34320 Avcilar, Istanbul, Turkey; selins@istanbul.edu.tr; 2 Department of Computer Engineering, Engineering Faculty, Istanbul University, 34320 Avcilar, Istanbul, Turkey; ruyasamli@istanbul.edu.tr; 3 Department of Chemical Pharmaceutical Microbiology, Faculty of Pharmacy, Istanbul University, 34116 Beyazıt, Istanbul, Turkey; seher.tan@istanbul.edu.tr; 4 Nutrition and Food Science Area, Preventive Medicine and Public Health, Food Science, Toxicology and Forensic Medicine Department, Faculty of Pharmacy, Universitat de València, Avda. Vicent Andrés Estellés, s/n, 46100 Burjassot, València, Spain; francisco.barba@uv.es; 5 Avignon University, INRA, Green Extraction Team, 84916 Avignon, France; farid.chemat@univ-avignon.fr; 6 Dipartimento di Scienza e Tecnologia del Farmaco, University of Turin, Via P. Giuria 9, 10125 Turin, Italy; 7 Centro Tecnológico de la Carne de Galicia, c/ Galicia, 4, 32900 San Ciprián de Viñas, Ourense, Spain; jmlorenzo@ceteca.net

**Keywords:** olive leaves, solvent-free microwave extraction, oleuropein, antioxidant, antimicrobial, optimization

## Abstract

Response surface methodology (RSM) and artificial neural networks (ANN) were evaluated and compared in order to decide which method was the most appropriate to predict and optimize total phenolic content (TPC) and oleuropein yields in olive tree leaf (*Olea europaea*) extracts, obtained after solvent-free microwave-assisted extraction (SFMAE). The SFMAE processing conditions were: microwave irradiation power 250–350 W, extraction time 2–3 min, and the amount of sample 5–10 g. Furthermore, the antioxidant and antimicrobial activities of the olive leaf extracts, obtained under optimal extraction conditions, were assessed by several in vitro assays. ANN had better prediction performance for TPC and oleuropein yields compared to RSM. The optimum extraction conditions to recover both TPC and oleuropein were: irradiation power 250 W, extraction time 2 min, and amount of sample 5 g, independent of the method used for prediction. Under these conditions, the maximal yield of oleuropein (0.060 ± 0.012 ppm) was obtained and the amount of TPC was 2.480 ± 0.060 ppm. Moreover, olive leaf extracts obtained under optimum SFMAE conditions showed antibacterial activity against *S. aureus* and *S. epidermidis*, with a minimum inhibitory concentration (MIC) value of 1.25 mg/mL.

## 1. Introduction

Olive trees come from a genus of evergreen trees in the family *Oleaceae*, which contains 24 genera and 900 species [1]. Nowadays, a great amount of wastes and by-products (e.g., crude olive cake, vegetation water, and twigs and leaves, which represent ≈10% of the total weight of the olives) are generated during the olive oil production process, fruit harvesting, and tree pruning [2,3].

Olive leaves and cakes have been traditionally used for animal feeding, but they can be used in other applications (e.g., food additives, nutraceuticals, and pharmaceutical and cosmetic purposes) due to their high content of high added-value compounds with antioxidant and antimicrobial properties (e.g., vitamins, polyphenols, minerals, etc.) [2,4,5]. In this regard, Bouaziz et al. [6] found extraordinary antioxidant potential for olive leaf and cake extracts, mainly attributed to their polyphenolic composition (e.g., oleuropein, luteolin, and hydroxytyrosol). Moreover, in another study, the use of olive leaf extracts was recommended due to their rich phenolic composition and consequent beneficial effects on health [7]. Thus, increased interest has been shown to the recovery of phenolic compounds from olive leaves [5,7,8].

Conventionally, polyphenol extraction from plant matrices has been carried out using maceration assisted by liquid solvents, thus requiring long extraction times [9,10,11,12]. Therefore, at this stage of development, the development of new techniques which can replace conventional extraction methods (e.g., effective extraction methods + low-cost raw materials) is of great importance, because they can reduce both extraction time and solvent consumption, thus representing an environmental and economical alternative [13,14,15]. Compared with other techniques, microwave (MW)-assisted extraction (MAE) has several advantages (e.g., shorter extraction time, reduced solvent consumption, and higher extraction rates). Moreover, MAE offers the possibility of obtaining products labelled as “green” according to environmental standards, with high quality and lower cost [16,17,18,19]. There are many factors affecting MAE extraction efficiency (e.g., MW power, extraction time, solvent type and composition, liquid to solid ratio, sample particle size, soaking time, and number of extraction cycles) [17,20]. Therefore, it is of paramount importance to find optimal MAE operating conditions in order to scale-up the process for commercial applications. With respect to the extraction of bioactive compounds from raw plant materials, MAE favours diffusion of thermolabile chemical content from the plant matrix by rupturing cell walls [21]. The mechanism of this process can be explained by three stages [22]: By increasing the temperature and/or irradiation power, the target solute is separated from the plant active sites. Then, the diffusion of the solutes from the raw material to the solvent occurs. Finally, the solute is transferred from the plant matrix to the solvent system.

Response surface methodology (RSM) is an important tool for developing, improving, and optimizing processes, as well as analysing response-independent variable interactions, and predicting the responses [12,23,24]. The artificial neural networks (ANN) technique, which is based on a computing system, is used for non-linear multivariate modelling, to estimate the response in the investigated ranges. However, there is a lack of information evaluating and comparing the usefulness of both methodologies to optimize polyphenol extraction yield from olive leaves. Recently, they have been evaluated and compared for modelling and optimizing innovative extraction processes [24], such as MAE of total polyphenolic content (TPC) from chokeberries as a function of extraction time, MW power, and ethanol concentration [25]. It was found that both techniques provided good predictions, although ANN gave better, more accurate results. To the best of our knowledge, there are no available reports evaluating the potential of solvent-free MAE (SFMAE) of TPC and oleuropein from olive leaves. Thus, the aim of this study was to examine solvent-free MAE (SFMAE) as a method applicable for the extraction of polyphenolic compounds and oleuropein from olive tree (*Olea europaea*) leaves. For this purpose, RSM and ANN methodologies were compared and used to evaluate and optimize the main extraction parameters (the amount of sample, extraction time, and MW power), in order to obtain maximum TPC and oleuropein yields in extracts obtained using SFMAE. Furthermore, the antioxidant (Cupric Ion Reducing Antioxidant Capacity (CUPRAC), 2,2′-Azino-*bis*-(3-ethylbenzothiazoline-6-sulfonic acid) Diammonium Salt (ABTS), and 2,2-diphenyl-1-picrylhydrazyl (DPPH)) and antimicrobial activities of extracts obtained under optimal conditions were also evaluated. MAE is an appropriate technology for much larger scales of industrial application owing to its rapid heating, easy scaling, little/no solvent necessity, and green and continuous nature [26]. Therefore, the findings of the present study also aim to contribute to the scaling-up of processes, depending on the optimization results of the lab-scale processes.

## 2. Results and Discussion

The results of experimental and mathematical studies of SFMAE of olive leaves are presented in Table 1. It shows the effects of solid mass, MW irradiation power, and extraction time on the TPC and oleuropein in olive leaf extracts obtained by SFMAE.

### 2.1. Evaluation and Comparison of RSM and ANN Methodologies to Optimize Total Phenolic Compound and Oleuropein Extraction Yields under SFMAE

RSM and ANN methods were applied for modelling and predicting TPC and oleuropein yields from olive leaves after SFMAE.

#### 2.1.1. Modelling of SFMAE Using RSM

The calculated models for TPC and oleuropein yield were both found to be significant when indicating the relationship between the independent and dependent parameters (Table 2). Analysis of variance (ANOVA) results show that the experimental data had a coefficient of determination (*R*^2^) of almost 0.84 for the calculated models, accounting for 84% of the results. The amount of sample was found to be the most significant (*p* < 0.05) variable for the SFMAE of TPC from olive leaves, followed by extraction time, and MW irradiation power. Regarding oleuropein, the second power of MW power was the most significant parameter, followed by the amount of sample, quadratic time, and power (Table 2).

Second-order equations derived for TPC and oleuropein are given in Equations (1) and (2), respectively (see Equation (1) Part 3.7.1.):

(1) $$\begin{array}{l} Y_{1}=-0.019369-0.36003X_{1}+0.14249X_{2}-13.61029X_{3}+6.64\times 10^{-4}X_{1}X_{2}+0.089174X_{1}X_{3}+\\ 4.53\times 10^{-3}X_{2}X_{3}-0.012889X_{1}^{2}-2.74\times 10^{-4}X_{2}^{2}+2.34993X_{3}^{2} \end{array}$$

(2) $$\begin{array}{l} Y_{2}=0.62767-0.029622X_{1}-2.60\times 10^{-3}X_{2}-0.056494X_{3}+4.26\times 10^{-5}X_{1}X_{2}+5.07\times 10^{-3}X_{1}X_{3}+\\ 2.48\times 10^{-4}X_{2}X_{3}+1.15\times 10^{-4}X_{1}^{2}+2.53\times 10^{-6}X_{2}^{2}-0.013423X_{3}^{2} \end{array}$$

where *X*_1_ is the amount of sample, *X*_2_ is the MW irradiation power, and *X*_3_ is the extraction time.

The independent and dependent parameters were also tested for lack of fit in the quadratic response surface models. The model derived for TPC was found to be insignificant (*p* > 0.05), verifying that the model could adequately fit the experimental data [27]. However, the model calculated for oleuropein yield was found to be significant (*p* < 0.05). Regardless, the results are available for inclusion in the analysis, taking into account recommendations from other authors. For instance, Kittisuban et al. [28] reported that a model with a significant lack of fit can be used when large amounts of data were included in the analysis. In this line, other previous works also found similar behaviour in plant matrices [29,30].

**Figure 1:**
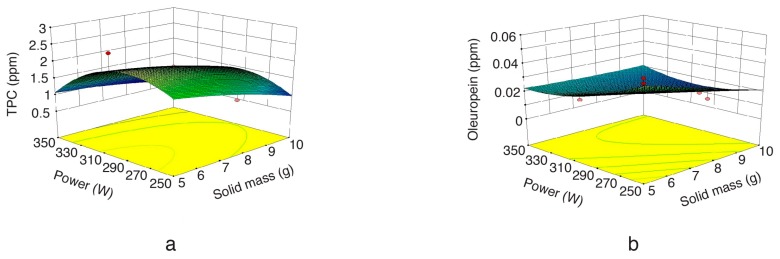
Response surface plot for the TPC (**a**) and oleuropein (**b**) from olive leaf extract as a function of microwave irradiation power to solid mass (extraction time = 2.5 min).

**Figure 2:**
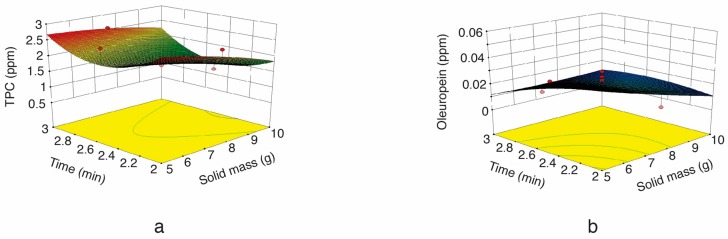
Response surface plot for the TPC (**a**) and oleuropein (**b**) from olive leaf extract as a function of extraction time to solid mass (microwave irradiation power = 300 W).

**Figure 3:**
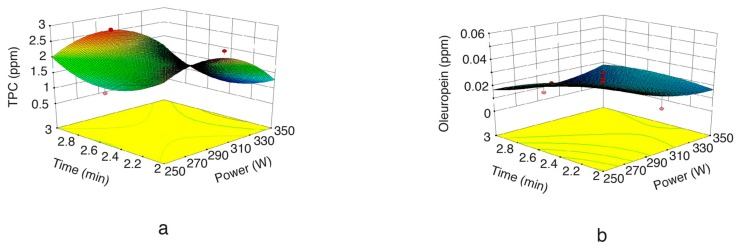
Response surface plot for the TPC (**a**) and oleuropein (**b**) from olive leaf extract as a function of extraction time to microwave irradiation power (solid mass = 7.5 g).

Three-dimensional response surface plots (Figure 1, Figure 2 and Figure 3) were developed according to Equations (2) and (3), to predict interactions among the different variables and their corresponding effect on the response variables. As can be seen in Figure 1a, an increase in MW power enhanced the extraction yield at first, and then decreased it. This outcome is consistent with the data reported by Hayat et al. [20] and Périno-Issartier et al. [31], who also observed a similar trend for the effect of MW irradiation power on the MAE of natural antioxidants from several plant materials. On the other hand, high MW irradiation power promoted an oleuropein yield decrease (Figure 1b). This fact can be attributed to overheating, which leads to the degradation of heat-sensitive oleuropein.

Regarding the impact of the amount of sample on the SFMAE of TPC and oleuropein, Figure 2a,b show decreased polyphenol extraction when the amount of sample was increased. A similar trend was reported by Ballard et al. [32], who also observed a significant reduction (35.8%) in TPC yield when the amount of peanut skin was increased from 1.5 to 3.5 g. This can be attributed to a decrease in surface area (increased sample amount and constant solvent volume), thus impairing solvent penetration into the matrix and the subsequent solubilization of TPC, thus promoting a reduced extraction yield.

Figure 3a,b indicate the effect of extraction time on TPC and oleuropein extraction. As can be seen in the figure, it was observed that extraction time had a negative influence on the SFMAE of TPC and oleuropein. This result might be explained by the fact that long extraction times promote oleuropein degradation, mainly due to overheating during long extraction times. TPC had a slight rise after a while, since heat sensitive materials might promote the degradation of other compounds; therefore, those components coming from degraded polyphenols could contribute to the formation of other polyphenols in the extract. Similarly, Ballard et al. [32] also observed a non-significant TPC trend, with a decrease in TPC extraction from peanut skins when irradiation time was augmented at 90% power. These results suggest that applying high MW powers for short times may be the most effective way to extract TPC from olive leaves.

#### 2.1.2. Modelling of SFMAE Using ANN

**Figure 4:**
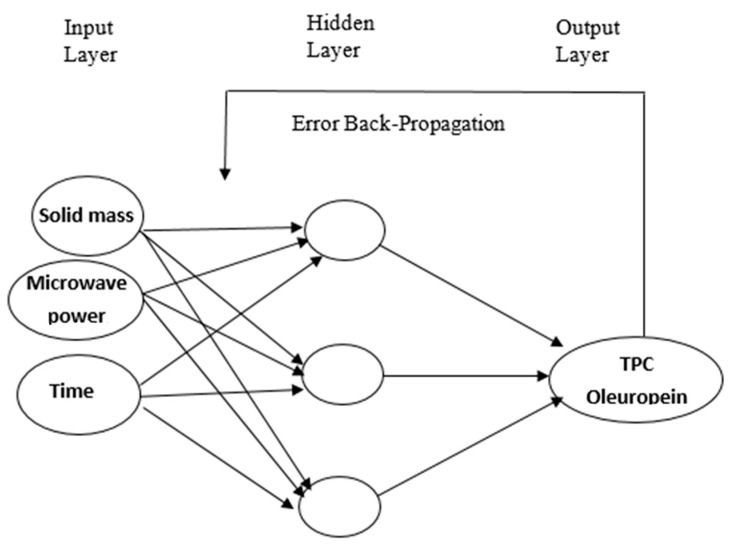
The feed-forward multilayer artificial neural network model with a back-propagation learning algorithm.

Figure 4 shows the structure of the feed-forward multilayer ANN model with a back-propagation learning algorithm, designed for the relevant process. The input layer represents vectors constituted of non-coded variables, and the output layer represents TPC and oleuropein. In the ANN structure, the unit number in the hidden layer was chosen as 10, based on many previous studies in the literature. The input data for training, validation, and the test phase were chosen as 70%, 15%, and 15%, respectively, and the Levenberg–Marquardt algorithm was used as the training algorithm.

The predicted values after ANN modelling are shown in Table 1.

Table 1 indicates the differences between the experimental and predicted results of RSM and ANN approaches for TPC and oleuropein, respectively. As can be seen in Table 1, both RSM and ANN models provided predictions of great quality when compared with the experimental values, although ANN showed better accuracy and generalization capabilities compared to RSM, despite the limited number of experiments. RSM is useful in obtaining insight information (such as interactions between components) for the system directly due to its natural properties, whereas ANN is useful in sensitivity analysis (prediction and estimation). Thus, it would be more rational and reliable to evaluate data regarding the SFMAE of TPC and oleuropein from olive leaves data through a process of ANN architecture.

#### 2.1.3. Antioxidant and Antimicrobial Activities of the Extracts Obtained from Olive Leaves at Optimal SFMAE Conditions

Taking into account the experimental results obtained, and the predicted and optimized values from ANN and RSM, the conditions selected to optimize, at the same time, the extraction of TPC and oleuropein through SFMAE were: MW power of 250 W, for 2 min, using 5 g of olive leaves. Under these conditions, the TPC and oleuropein concentrations in the extract were 2.50 and 0.06 ppm, respectively.

The antioxidant capacity of the extracts differed according to the method used, obtaining the highest antioxidant value (1700 µg TEAC/g dry leaves) when a CUPRAC assay was used, compared to the values obtained for ABTS (580 µg TEAC/g dry leaves), and DPPH (20 µg TEAC/g dry leaves) assays. This fact can be attributed to the different antioxidant compounds found in the samples (vitamins C, E, chlorophylls, carotenoids, polyphenols, etc.) having different roles as antioxidants [33]. In line with the results obtained in the present study, Li et al. [34] also found higher antioxidant values with CUPRAC compared to DPPH and ABTS methods, in grape seed powder and China wines.

Moreover, the antimicrobial potential of the extracts was also examined. In this study, ciprofloxacin and fluconazole were used as reference antimicrobials for bacteria and yeast, respectively. Furthermore, the antimicrobial effects of DMSO were also investigated against test microorganisms, as a control. The results were evaluated according to the values of the control (data not shown). Ciprofloxacin and fluconazole MIC values were within the accuracy range for the CLSI throughout the study [35]. Olive leaf extract obtained at SFMAE optimum conditions showed antibacterial activity against *S. aureus* and *S. epidermidis*, with a MIC value of 1250 µg/mL. This outcome is in close agreement with the data previously reported by Pereira et al. [7], who also found important concentration-dependent antimicrobial activity of olive leaf extracts against bacteria and fungi. They attributed the antimicrobial activity to oleuropein and TPC identified in the extract [7].

## 3. Material and Methods

### 3.1. Plant Material

Fresh olive leaves were harvested from Ayvalik (Edremit) cultivar growing in the Aegean part of Turkey during the harvesting time of olives (November 2015), and provided by Özgün Olive, Olive Oil Co.’s (Turkey) relevant departments. They were collected, dried, and stored at 4 °C, until needed for extraction. Prior to extraction, olive leaves were ground into particles (diameter ≈ 0.9–2.0 mm).

### 3.2. Chemicals and Reagents

Ethanol and methanol were provided from Merck (Darmstadt, Germany). Eighteen mΩ deionised water (from a Human Power I water purification system) was used for analysis. Oleuropein, 2,9-dimethyl-1,10-phenanthroline (neocuproine), 6-hydroxy-2,5,7,8-tetramethylchroman-2-carboxylic acid (Trolox^®^), Folin–Ciocalteu reagent, sodium carbonate, gallic acid, 2,2-diphenyl-1-picrylhydrazyl (DPPH), and 2,2′-azino-*bis*-(3-ethylbenzothiazoline-6-sulfonic acid) diammonium salt (ABTS), were purchased from Sigma-Aldrich (St. Louis, MO, USA).

### 3.3. Solvent-Free Microwave-Assisted Extraction (SFMAE)

**Figure 5:**
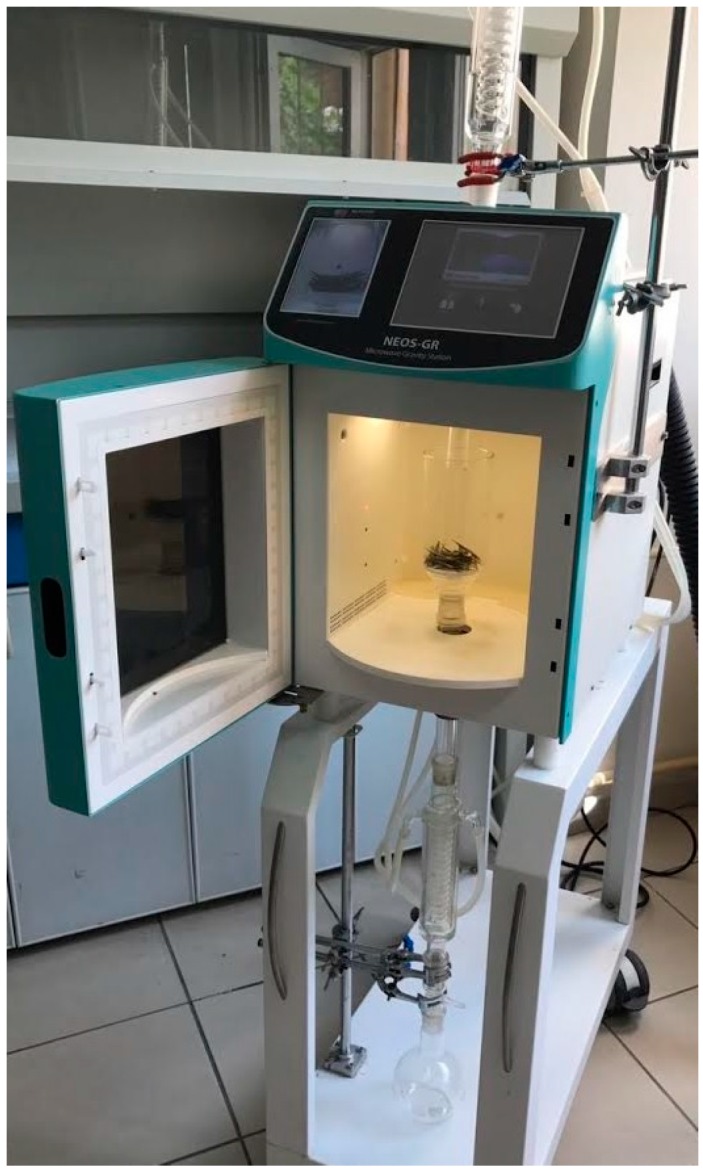
Reactor for solvent-free microwave-assisted extraction.

As seen in Figure 5, the equipment for SFMAE consists of MW apparatus (NEOS-GR, Milestone Srl, Italy) operating at 2.45 GHz (900 W maximum power). The extractor had specific software and a video camera. Water was used as pre-treatment to enhance the extraction process. Olive leaves (5, 7.5, and 10 g) were placed in a 1 L Pyrex^®^ glass beaker and placed in the MW oven at 250, 300, and 350 W for a certain period of time (2, 2.5, and 3 min). The extraction was performed at atmospheric pressure. The extract was drained by gravity on a condenser outside the MW irradiation cavity and was cooled down at room temperature [36]. Then, the mixture was centrifuged (Nuve, CN 180) at 5000× *g* for 25 min. Subsequently, the supernatant was filtered through a 0.45 μm syringe filter and stored at −80 °C until needed for analysis.

### 3.4. Identification and Quantification of Polyphenols

#### 3.4.1. TPC

The concentration of TPC in olive leaf extracts was measured by UV-spectrophotometry (PG Instruments, T60/Leicestershire, England, UK), based on a colorimetric oxidation/reduction reaction. The TPC was determined according to the Folin–Ciocalteau method proposed by Malik and Bradford [37]. The amount of TPC was expressed in mg of gallic acid equivalent per kg of dried leaf (ppm or mg/kg).

#### 3.4.2. HPLC-DAD Identification and Quantification of Oleuropein

**Figure 6:**
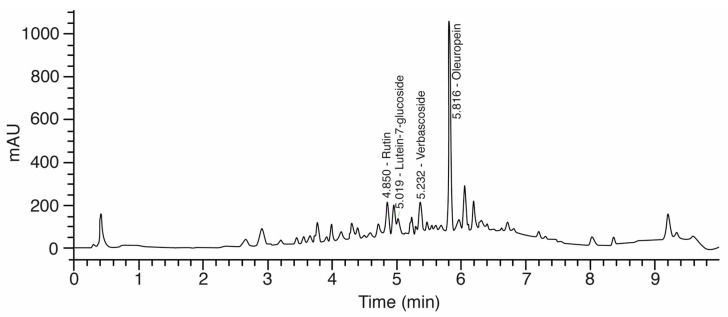
A typical chromatogram of individual polyphenol composition in olive leaf extracts.

Oleuropein content was determined according to the method proposed by Guinda et al. [38] with some minor modifications. Agilent 1260 chromatographic equipment (Agilent, Waldbronn, Germany) equipped with a quaternary pump, a degasser, a manual injector, and a diode-array detector (DAD) was used. An Agilent Eclipse Plus C18 RRHD 18 column (3.0 mm × 5.0 mm id, 1.8 μm particle size) was used for the analyses. The temperature of the column was kept at 40 °C through a gradient elution of (A) 0.1% formic acid in H_2_O and (B) 0.1% formic acid in acetonitrile. A gradient program was written according to the following profile: 0–7 min 0% B, 7–7.1 min 40% B, 7.1–8.6 min 100% B, and 8.6–10 min 0% B. Injection volume was 20 μL and the detection wavelength was set at 276 nm. A typical chromatogram is shown in Figure 6.

### 3.5. Antioxidant Activity

#### 3.5.1. 2,2-Diphenyl-1-picrylhydrazyl (DPPH) Assay

The DPPH assay was conducted according to the method established by the authors of [39] with some minor modifications. Samples were diluted with a methanolic solution of DPPH radical (500 µM) to obtain a final concentration of 100 µM. Then, they were vigorously mixed and kept under darkness conditions for 30 min at 25 °C. The absorbance was measured at 517 nm against a blank sample without DPPH. Results were expressed as mg trolox equivalent (TE)/g of dried leaf.

#### 3.5.2. 2,2′-Azino-*bis*-(3-ethylbenzothiazoline-6-sulfonic acid) Diammonium Salt (ABTS) Assay [40]

The ABTS assay was performed according to the method of Re et al. [27]. One hundred and fifty microliters of sample solution was added into 2850 μL of diluted ABTS solution. Then, the absorbance was measured after 10 min at 734 nm against a blank sample (without ABTS). Results were expressed as mg trolox equivalent antioxidant capacity (TEAC)/g of dried leaf.

#### 3.5.3. Cupric Ion Reducing Antioxidant Capacity (CUPRAC) Assay

The CUPRAC method of Apak et al. [41] was also applied to the olive leaf extracts. The absorbance of the extract samples was measured at 450 nm against a blank sample without CUPRAC. Results were expressed as mg trolox equivalent antioxidant capacity (TEAC)/g of dried leaf.

### 3.6. Antimicrobial Activity

The in vitro antimicrobial activities of the extracts were determined using the microbroth dilution technique described by the Clinical and Laboratory Standards Institute [42]. The minimum inhibitory concentrations (MICs) of the extracts were evaluated against *Staphylococcus aureus* ATCC 29213, *Staphylococcus epidermidis* ATCC 12228, *Escherichia coli* ATCC 25922, *Enterococcus faecalis* ATCC 29212, *Klebsiella pneumoniae* ATCC 4352, *Pseudomonas aeruginosa* ATCC 27853, *Proteus mirabilis* ATCC 14153, and *Candida albicans* ATCC 10231. Serial 2-fold dilutions (from 5000 to 4.8 μg/mL) were prepared in Mueller–Hinton broth (MHB) (Difco, Detroit, MI, USA) for the bacteria, and RPMI-1640 medium buffered to pH 7.0 with MOPS was prepared for the yeast strain, as test media (Sigma, St. Louis, MO, USA). Dimethyl sulfoxide (DMSO) was used as a solvent for the extracts. Each well was inoculated with 50 μL of a 4–6 h broth culture to give a final concentration of 5 × 10^5^ cfu/mL for the bacteria, and 0.5 × 10^3^ to 2.5 × 10^3^ cfu/mL for the yeast, in the test trays, respectively. The trays were covered and placed in plastic bags to prevent evaporation. The trays containing MHB were incubated at 37 °C for 24 h, while those containing the RPMI-1640 medium were incubated at 30 °C for 48 h. The MIC of each extract was defined as the lowest concentration of compound required for complete inhibition of visible growth.

### 3.7. Experimental Design for Optimization and Statistical Analysis

#### 3.7.1. Response Surface Methodology (RSM)

A face central composite design (FCCD) was carried out with the three independent parameters to find out the effect of the parameters on the response variables (Table 1). The TPC (*Y*_1_) and oleuropein (*Y*_2_) were chosen as the response variables. Amount of sample (X_1_), MW irradiation power (X_2_), and extraction time (X_3_) were the independent variables, selected based on preliminary experiments including three levels of each independent variable. Twenty experiments were conducted, with six replications at the center values to evaluate the pure error sum of squares. Experimental data were fitted to the quadratic model. The quadratic model proposed is shown as follows in Equation (3):

(3) $$Y=\overset{n}{\underset{i=1}{\sum }}\beta_{i}X_{i}+\sum \overset{n}{\underset{i<j}{\sum }}\beta_{ij}X_{i}X_{j}+\sum \sum \overset{n}{\underset{i<j<k}{\sum }}\beta_{ijk}X_{i}X_{j}X_{k}$$

where *Y* is the dependent parameter, β_0_ is the constant coefficient known as intercept, *X_i_* (*i* = 1–3) is the non-coded variable, and β*_i_,* β*_ii_,* and β*_ij_* (*i* and *j* = 3) are the linear, quadratic, and interception coefficients, respectively.

#### 3.7.2. Artificial Neural Network (ANN)

A three-layered feed-forward back-propagation neural network with a linear transfer function was developed for modelling TPC and oleuropein extraction from olive leaves. ANN consisted of three layers: (i) an input layer (independent variables: sample (g), MW power (W), and extraction time (min)), (ii) an output layer (dependent variables: TPC and oleuropein), and (iii) one or more intermediate nodes (a collection of feature detectors) [43]. Taking into account the selected independent and dependent variables, a network with three neurons in the input layer and one in the output layer was the selected model. Then, the best neural network model was generated following a number of training trials.

For both RSM and ANN methods, three replicates of each extraction were carried out for each of the samples, followed by a minimum of three spectrophotometric and HPLC measurements from each extract. Design Expert (Trial version 8.0.6.2) statistical software was used to analyze the experimental data for an analysis of variance (ANOVA).

## 4. Conclusions

In this study, two different types of modelling methods (RSM and ANN) were applied to study, predict, and estimate the behaviour of TPC and oleuropein in olive leaf extracts obtained by SFMAE. The quadratic models generated by RSM design were found to be suitable for predicting the TPC and oleuropein amounts in every experimental study. The designed ANN model could predict the minimum/maximum values and increment/decrement tendency of the responses. RSM showed higher deviation than ANN for modelling TPC and oleuropein extraction from olive leaves assisted by SFMAE. From both predictive methods, it can be concluded that MW power of 250 W, for 2 min, and 5 g of olive leaf extract, were the optimal processing conditions to extract TPC and oleuropein, obtaining 2.50 and 0.06 ppm of TPC and oleuropein, respectively, under these conditions. Moreover, it can also be concluded that SFMAE is scalable [44], and olive leaf extracts obtained by optimized conditions can be used in the food industry (food additives, nutraceuticals, etc.), exploiting their antioxidant and antimicrobial activities.

## Figures and Tables

**Table 1:** A comparison between the experimental and predicted values obtained for TPC and oleuropein yields after solvent-free microwave-assisted extraction using response surface methodology (RSM) and artificial neural networks (ANN).

No.	*X*_1_ (g)	*X*_2_ (W)	*X*_3_ (min)	TPC Amount (ppm)		
				Experimental	RSM Predicted	ANN Predicted
1	10	350	3	1.46 ± 0.04	0.44	1.27
2	5	250	2	2.48 ± 0.06	2.06	2.74
3	5	350	2	1.32 ± 0.04	1.11	1.51
4	7.5	300	2.5	1.72 ± 0.01	1.15	1.02
5	5	350	3	1.61 ± 0.01	2.04	1.94
6	7.5	350	2.5	1.30 ± 0.03	0.18	1.01
7	7.5	300	3	2.57 ± 0.03	1.50	2.58
8	7.5	300	2.5	1.82 ± 0.01	1.15	1.11
9	7.5	250	2.5	1.30 ± 0.01	0.74	1.32
10	10	250	3	1.84 ± 0.01	0.61	1.77
11	5	300	2.5	2.62 ± 0.03	1.40	2.47
12	5	250	3	2.17 ± 0.02	1.20	2.24
13	10	250	2	1.56 ± 0.02	1.02	1.40
14	7.5	300	2.5	1.85 ± 0.02	1.15	1.78
15	7.5	300	2	2.58 ± 0.04	1.19	2.76
16	7.5	300	2.5	1.75 ± 0.04	1.15	1.20
17	7.5	300	2.5	1.82 ± 0.01	1.15	1.87
18	10	300	2.5	1.19 ± 0.01	0.74	1.34
19	10	350	2	0.87 ± 0.00	0.40	1.16
20	7.5	300	2.5	1.80 ± 0.00	1.15	1.77
**No.**	***X*_1_ (g)**	***X*_2_ (W)**	***X*_3_ (min)**	**Oleuropein Amount (ppm)**		
				**Experimental**	**RSM Predicted**	**ANN Predicted**
1	10	350	3	0.0264 ± 0.00	0.0142	0.0272
2	5	250	2	0.0596 ± 0.01	0.0518	0.0592
3	5	350	2	0.0286 ± 0.00	0.0145	0.0278
4	7.5	300	2.5	0.0234 ± 0.00	0.0115	0.0242
5	5	350	3	0.0100 ± 0.00	0.0031	0.0103
6	7.5	350	2.5	0.0196 ± 0.00	0.0107	0.0208
7	7.5	300	3	0.0123 ± 0.00	0.0025	0.0125
8	7.5	300	2.5	0.0299 ± 0.00	0.0115	0.0242
9	7.5	250	2.5	0.0256 ± 0.00	0.0249	0.0372
10	10	250	3	0.0114 ± 0.00	0.0054	0.0235
11	5	300	2.5	0.0249 ± 0.01	0.0183	0.0251
12	5	250	3	0.0252 ± 0.00	0.0156	0.0595
13	10	250	2	0.0294 ± 0.00	0.0163	0.0316
14	7.5	300	2.5	0.0261 ± 0.00	0.0115	0.0254
15	7.5	300	2	0.0134 ± 0.00	0.0137	0.0150
16	7.5	300	2.5	0.0202 ± 0.01	0.0115	0.0205
17	7.5	300	2.5	0.0200 ± 0.00	0.0115	0.0200
18	10	300	2.5	0.0090 ± 0.00	0.0061	0.0097
19	10	350	2	0.0108 ± 0.00	0.0029	0.0117
20	7.5	300	2.5	0.0200 ± 0.01	0.0115	0.0211

**Table 2:** ANOVA for the quadratic equations for solvent-free microwave-assisted extraction (SFMAE) of TPC and oleuropein from olive leaves.

Source	Sum of Squares	df	Mean Square	*F-*Value	*p*-Value Prob > F
Model (TPC)	2.006 × 10^−3^	9	2.229 × 10^−4^	5.95	0.0051
X_1_	3.751 × 10^−4^	1	3.751 × 10^−4^	10.01	0.0101
X_2_	3.119 × 10^−4^	1	3.119 × 10^−4^	8.32	0.0162
X_3_	3.202 × 10^−4^	1	3.202 × 10^−4^	8.55	0.0152
X_1_X_2_	2.266 × 10^−4^	1	2.266 × 10^−4^	6.05	0.0337
X_1_X_3_	3.215 × 10^−4^	1	3.215 × 10^−4^	8.58	0.0151
X_2_X_3_	3.064 × 10^−4^	1	3.064 × 10^−4^	8.18	0.0170
X_1_^2^	1.412 × 10^−6^	1	1.412 × 10^−6^	0.038	0.8500
X_2_^2^	1.103 × 10^−4^	1	1.103 × 10^−4^	2.94	0.1170
X_3_^2^	3.097 × 10^−5^	1	3.097 × 10^−5^	0.83	0.3847
Residual	3.747 × 10^−4^	10	3.747 × 10^−5^		
Lack of Fit	2.920 × 10^−4^	5	5.840 × 10^−5^	3.53	0.0962
Pure Error	8.269 × 10^−5^	5	1.654 × 10^−5^		
Cor Total	2.381 × 10^−3^	19			
Model (oleuropein)	3.92	9	0.44	6.00	0.0049
X_1_	1.08	1	1.08	14.85	0.0032
X_2_	0.78	1	0.78	10.72	0.0084
X_3_	0.069	1	0.069	0.95	0.3528
X_1_X_2_	0.055	1	0.055	0.76	0.4042
X_1_X_3_	0.099	1	0.099	1.37	0.2690
X_2_X_3_	0.10	1	0.10	1.41	0.2623
X_1_^2^	0.018	1	0.018	0.25	0.6307
X_2_^2^	1.29	1	1.29	17.77	0.0018
X_3_^2^	0.95	1	0.95	13.08	0.0047
Residual	0.73	10	0.073		
Lack of Fit	0.71	5	0.14	54.91	0.0002
Pure Error	0.013	5	2.596 × 10^−3^		
Cor Total	4.64	19			

## Supplementary Materials

Supplementary File 1

## References

[1] Ghazanfar S.A., Ghazanfar S.A. (1994). Handbook of Arabian Medicinal Plants.

[2] Roselló-Soto E., Koubaa M., Moubarik A., Lopes R.P., Saraiva J.A., Boussetta N., Grimi N., Barba F.J. (2015). Emerging opportunities for the effective valorization of wastes and by-products generated during olive oil production process: Non-conventional methods for the recovery of high-added value compounds. Trends Food Sci. Technol..

[3] Roselló-Soto E., Barba F.J., Parniakov O., Galanakis C.M., Lebovka N., Grimi N., Vorobiev E. (2015). High Voltage Electrical Discharges, Pulsed Electric Field, and Ultrasound Assisted Extraction of Protein and Phenolic Compounds from Olive Kernel. Food Bioprocess Technol..

[4] Granato D., Nunes D.S., Barba F.J. (2017). An integrated strategy between food chemistry, biology, nutrition, pharmacology, and statistics in the development of functional foods: A proposal. Trends Food Sci. Technol..

[5] Moudache M., Colon M., Nerín C., Zaidi F. (2016). Phenolic content and antioxidant activity of olive by-products and antioxidant film containing olive leaf extract. Food Chem..

[6] Bouaziz M., Fki I., Jemai H., Ayadi M., Sayadi S. (2008). Effect of storage on refined and husk olive oils composition: Stabilization by addition of natural antioxidants from Chemlali olive leaves. Food Chem..

[7] Pereira A.P., Ferreira I.C., Marcelino F., Valentão P., Andrade P.B., Seabra R., Estevinho L., Bento A., Pereira J.A. (2007). Phenolic compounds and antimicrobial activity of olive (*Olea europaea* L. Cv. Cobrançosa) leaves. Molecules.

[8] Ahmad-Qasem M.H., Cánovas J., Barrajón-Catalán E., Micol V., Cárcel J.A., García-Pérez J.V. (2013). Kinetic and compositional study of phenolic extraction from olive leaves (var. Serrana) by using power ultrasound. Innov. Food Sci. Emerg. Technol..

[9] Barba F.J., Zhu Z., Koubaa M., Sant’Ana A.S., Orlien V. (2016). Green alternative methods for the extraction of antioxidant bioactive compounds from winery wastes and by-products: A review. Trends Food Sci. Technol..

[10] Roselló-Soto E., Parniakov O., Deng Q., Patras A., Koubaa M., Grimi N., Boussetta N., Tiwari B.K., Vorobiev E., Lebovka N. (2016). Application of Non-conventional Extraction Methods: Toward a Sustainable and Green Production of Valuable Compounds from Mushrooms. Food Eng. Rev..

[11] Koubaa M., Rosello-Sotó E., Šic Žlabur J., Režek Jambrak A., Brnčić M., Grimi N., Boussetta N., Barba F.J. (2015). Current and new insights in the sustainable and green recovery of nutritionally valuable compounds from Stevia rebaudiana Bertoni. J. Agric. Food Chem..

[12] Putnik P., Bursać Kovačević D., Dragović-Uzelac V. (2016). Influence of Acidity and Extraction Time on the Recovery of Flavonoids from Grape Skin Pomace Optimized by Response Surface Methodology. Chem. Biochem. Eng. Q..

[13] Chemat F., Fabiano-Tixier A.S., Abert Vian M., Allaf T., Vorobiev E. (2017). Solvent-Free Extraction. Compr. Anal. Chem..

[14] Baghdikian B., Filly A., Fabiano-Tixier A.-S., Petitcolas E., Mabrouki F., Chemat F., Ollivier E. (2016). Extraction by solvent using microwave and ultrasound-assisted techniques followed by HPLC analysis of Harpagoside from Harpagophytum procumbens and comparison with conventional solvent extraction methods. C. R. Chim..

[15] Bilgin M., Şahin S. (2013). Effects of geographical origin and extraction methods on total phenolic yield of olive tree (*Olea europaea*) leaves. J. Taiwan Inst. Chem. Eng..

[16] Putnik P., Kovačević D.B., Penić M., Fegeš M., Dragović-Uzelac V. (2016). Microwave-assisted extraction (MAE) of dalmatian sage leaves for the optimal yield of polyphenols: HPLC-DAD identification and quantification. Food Anal. Methods.

[17] Périno S., Pierson J.T., Ruiz K., Cravotto G., Chemat F. (2016). Laboratory to pilot scale: Microwave extraction for polyphenols lettuce. Food Chem..

[18] Li Y., Fabiano-Tixier A.S., Vian M.A., Chemat F. (2013). Solvent-free microwave extraction of bioactive compounds provides a tool for green analytical chemistry. TrAC-Trends Anal. Chem..

[19] Li Y., Skouroumounis G.K., Elsey G.M., Taylor D.K. (2011). Microwave-assistance provides very rapid and efficient extraction of grape seed polyphenols. Food Chem..

[20] Hayat K., Hussain S., Abbas S., Farooq U., Ding B., Xia S., Jia C., Zhang X., Xia W. (2009). Optimized microwave-assisted extraction of phenolic acids from citrus mandarin peels and evaluation of antioxidant activity in vitro. Sep. Purif. Technol..

[21] Ameer K., Shahbaz H.M., Kwon J.H. (2017). Green Extraction Methods for Polyphenols from Plant Matrices and Their Byproducts: A Review. Compr. Rev. Food Sci. Food Saf..

[22] Azmir J., Zaidul I.S.M., Rahman M.M., Sharif K.M., Mohamed A., Sahena F., Jahurul M.H.A., Ghafoor K., Norulaini N.A.N., Omar A.K.M. (2013). Techniques for extraction of bioactive compounds from plant materials: A review. J. Food Eng..

[23] Putnik P., Bursać Kovačević D., Dragović-Uzelac V. (2016). Optimizing Acidity and Extraction Time for Polyphenolic Recovery and Antioxidant Capacity in Grape Pomace Skin Extracts with Response Surface Methodology Approach. J. Food Process. Preserv..

[24] Roselló-Soto E., Galanakis C.M., Brnćić M., Orlien V., Trujillo F.J., Mawson R., Knoerzer K., Tiwari B.K., Barba F.J. (2015). Clean recovery of antioxidant compounds from plant foods, by-products and algae assisted by ultrasounds processing. Modeling approaches to optimize processing conditions. Trends Food Sci. Technol..

[25] Simić V.M., Rajković K.M., Stojičević S.S., Veličković D.T., Nikolić N.Č., Lazić M.L., Karabegović I.T. (2016). Optimization of microwave-assisted extraction of total polyphenolic compounds from chokeberries by response surface methodology and artificial neural network. Sep. Purif. Technol..

[26] Balasubramanian S., Allen J.D., Kanitkar A., Boldor D. (2011). Oil extraction from *Scenedesmus obliquus* using a continuous microwave system-design, optimization, and quality characterization. Bioresour. Technol..

[27] Silva E.M., Rogez H., Larondelle Y. (2007). Optimization of extraction of phenolics from *Inga edulis* leaves using response surface methodology. Sep. Purif. Technol..

[28] Kittisuban P., Ritthiruangdej P., Suphantharika M. (2014). Optimization of hydroxypropylmethylcellulose, yeast β-glucan, and whey protein levels based on physical properties of gluten-free rice bread using response surface methodology. LWT-Food Sci. Technol..

[29] Saldaña-Robles A., Guerra-Sánchez R., Maldonado-Rubio M.I., Peralta-Hernández J.M. (2014). Optimization of the operating parameters using RSM for the Fenton oxidation process and adsorption on vegetal carbon of MO solutions. J. Ind. Eng. Chem..

[30] Danish M., Hashim R., Ibrahim M.N.M., Sulaiman O. (2014). Optimized preparation for large surface area activated carbon from date (*Phoenix dactylifera* L.) stone biomass. Biomass Bioenerg..

[31] Périno-Issartier S., Zill-e-Huma, Abert-Vian M., Chemat F. (2011). Solvent free microwave-assisted extraction of antioxidants from Sea buckthorn (*Hippophae rhamnoides*) food by-products. Food Bioprocess Technol..

[32] Ballard T.S., Mallikarjunan P., Zhou K., O’Keefe S. (2010). Microwave-assisted extraction of phenolic antioxidant compounds from peanut skins. Food Chem..

[33] Barba F.J., Esteve M.J., Tedeschi P., Brandolini V., Frígola A. (2013). A comparative study of the analysis of antioxidant activities of liquid foods employing spectrophotometric, fluorometric, and chemiluminescent methods. Food Anal. Methods.

[34] Li H., Wang X., Li Y., Li P., Wang H. (2009). Polyphenolic compounds and antioxidant properties of selected China wines. Food Chem..

[35] Jena J., Debata N.K., Sahoo R.K., Subudhi E. (2015). Phylogenetic study of metallo-β-lactamase producing multidrug resistant *Pseudomonas aeruginosa* isolates from burn patients. Burns.

[36] Şahin S. (2015). A novel technology for extraction of phenolic antioxidants from mandarin (*Citrus deliciosa* Tenore) leaves: Solvent-free microwave extraction. Korean J. Chem. Eng..

[37] Malik N.S.A., Bradford J.M. (2006). Changes in oleuropein levels during differentiation and development of floral buds in “Arbequina” olives. Sci. Hortic. (Amst.).

[38] Guinda A., Castellano J.M., Santos-Lozano J.M., Delgado-Hervás T., Gutiérrez-Adánez P., Rada M. (2015). Determination of major bioactive compounds from olive leaf. LWT-Food Sci. Technol..

[39] Yu L.L., Zhou K.K., Parry J. (2005). Antioxidant properties of cold-pressed black caraway, carrot, cranberry, and hemp seed oils. Food Chem..

[40] Re R., Pellegrini N., Proteggente A., Pannala A., Yang M., Rice-Evans C. (1999). Antioxidant activity applying an improved ABTS radical cation decolorization assay. Free Radic. Biol. Med..

[41] Apak R., Güçlü K., Özyürek M., Çelik S.E. (2008). Mechanism of antioxidant capacity assays and the CUPRAC (cupric ion reducing antioxidant capacity) assay. Microchim. Acta.

[42] Jorgensen J.H., Hindler J.F., Reller L.B., Weinstein M.P. (2007). New consensus guidelines from the Clinical and Laboratory Standards Institute for antimicrobial susceptibility testing of infrequently isolated or fastidious bacteria. Clin. Infect. Dis..

[43] Haykin S., Haykin S. (1998). Neural Networks: A Comprehensive Foundation.

[44] Filly A., Fernandez X., Minuti M., Visinoni F., Cravotto G., Chemat F. (2014). Solvent Free Microwave Extraction of Essential Oil from Aromatic Herbs. From laboratory to pilot and industrial scale. Food Chem..

