# Supplementary material for: Solvent-Free Microwave-Assisted Extraction of Polyphenols from Olive Tree Leaves: Antioxidant and Antimicrobial Properties
Source: Molecules. 2017 Jun 24;22(7):1056. doi: 10.3390/molecules22071056 (PMC6152306; doi:10.3390/molecules22071056)
Supplement: Supplementary File 1 [file molecules-22-01056-s001.pdf]

Article

Solvent-free Microwave-assisted Extraction of Polyphenols from Olive Tree Leaves: Antioxidant and Antimicrobial Properties

Selin Şahin[1], Ruya Samli[1], Ayşe Seher Birteksöz Tan[2], Francisco J. Barba[3], Farid Chemat[4], Giancarlo Cravotto[5]*, and José M. Lorenzo[6]

SUPPORTING INFORMATION

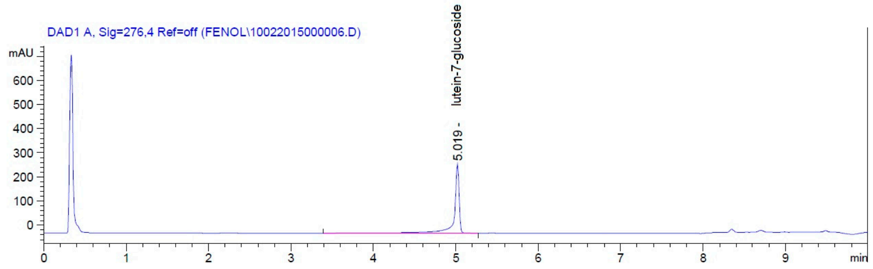

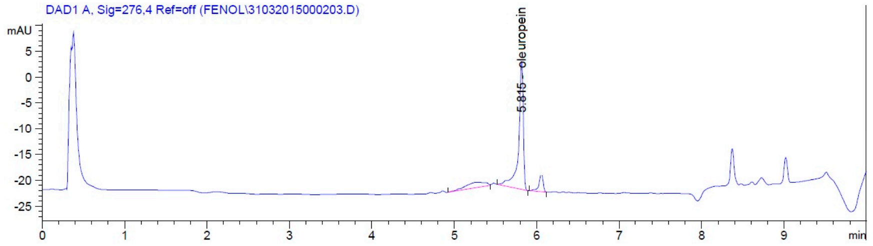

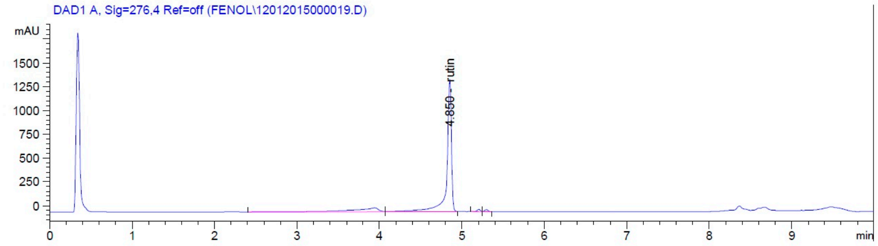
DAD1 A, Sig=276,4 Ref=off (FENOL\12012015000019.D)
mAU
4.850 - rutin
min

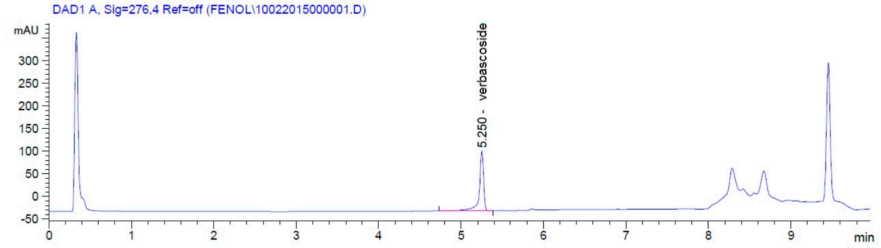
DAD1 A, Sig=276,4 Ref=off (FENOL\10022015000001.D)
mAU
5.250 - verbascoside
min
